# COVID-19 in three waves in a tertiary referral hospital in Belgium: a comparison of patient characteristics, management, and outcome

**DOI:** 10.1186/s12985-024-02360-8

**Published:** 2024-05-30

**Authors:** Andreas De Paepe, Erika Vlieghe, Nele Brusselaers, Patrick Soentjens, Caroline Theunissen, Isabel Brosius, Jeroen Grouwels, Lida Van Petersen, Hanne van Tiggelen, Walter Verbrugghe, Philippe G Jorens, Thérèse Lapperre, Karen Peeters, Griet Vermeulen, Sabrina H van Ierssel

**Affiliations:** 1 Department of General Internal Medicine, Infectious Diseases, and Tropical Medicine, Antwerp University Hospital, Drie Eikenstraat 655, Edegem, 2650 Belgium; 2 Global Health Institute, Department of Family Medicine and Population Health, University of Antwerp, Antwerp, Belgium; 3 Department of Public Health & Primary Care, Ghent University, Ghent, Belgium; 4 Centre for Translational Microbiome Research, Department of Microbiology Tumor and Cell Biology, Karolinska Institutet, Stockholm, Sweden; 5 Department of Clinical Sciences, Institute of Tropical Medicine, Antwerp, Belgium; 6 Clinical Research Center, Antwerp University Hospital, Edegem, Belgium; 7 Department of Intensive Care, Antwerp University Hospital, Edegem, Belgium; 8 Translational Research in Immunology and Inflammation, Laboratory of Experimental Medicine and Pediatrics, University of Antwerp, Antwerp, Belgium; 9 Department of Pneumology, Antwerp University Hospital, Edegem, Belgium; 10 Department of Emergency Medicine, Antwerp University Hospital, Edegem, Belgium; 11 Antwerp Surgical Training, Anatomy and Research Centre, University of Antwerp, Antwerp, Belgium

**Keywords:** COVID-19, Comparative analysis, Epidemic waves, Retrospective cohort study, Multivariable logistic regression, Intensive care unit subgroup analysis.

## Abstract

**Purpose:**

Few studies have compared patient characteristics, clinical management, and outcome of patients with COVID-19 between the different epidemic waves. In this study, we describe patient characteristics, treatment, and outcome of patients admitted for COVID-19 in the Antwerp University Hospital over the first three epidemic waves of 2020–2021.

**Methods:**

Retrospective observational study of COVID-19 patients in a Belgian tertiary referral hospital. All adult patients with COVID-19, hospitalized between February 29, 2020, and June 30, 2021, were included. Standardized routine medical data was collected from patient records. Risk factors were assessed with multivariable logistic regression.

**Results:**

We included 722 patients, during the first (*n* = 179), second (*n* = 347) and third (*n* = 194) wave. We observed the lowest disease severity at admission during the first wave, and more elderly and comorbid patients during the second wave. Throughout the subsequent waves we observed an increasing use of corticosteroids and high-flow oxygen therapy. In spite of increasing number of complications throughout the subsequent waves, mortality decreased each wave (16.6%,15.6% 11.9% in 1st, 2nd and 3rd wave respectively). C-reactive protein above 150 mg/L was predictive for the need for intensive care unit admission (odds ratio (OR) 3.77, 95% confidence interval (CI) 2.32–6.15). A Charlson comorbidity index ≥ 5 (OR 5.68, 95% CI 2.54–12.70) and interhospital transfers (OR 3.78, 95% CI 2.05–6.98) were associated with a higher mortality.

**Conclusions:**

We observed a reduction in mortality each wave, despite increasing comorbidity. Evolutions in patient management such as high-flow oxygen therapy on regular wards and corticosteroid use may explain this favorable evolution.

**Supplementary Information:**

The online version contains supplementary material available at 10.1186/s12985-024-02360-8.

## Background

Since December 2019, the SARS-CoV-2 pandemic has spread across the world. In Belgium, a first epidemic wave unfolded during spring 2020, a second in autumn and winter of 2020–2021, and a third wave spring 2021, coinciding with the emergence of the Alpha variant, lineage B.1.1.7 [1, 2]. 

Few studies have compared patient characteristics, treatments, and outcomes of patients with COVID-19 between these different epidemic waves [3–7]. Those who did, observed slightly older patients during the second pandemic wave, and a higher mortality during the first wave [3, 5–8], while one study observed lower mortality during the first wave, but fewer hospitalizations [4]. Higher mortality was observed in older patients, men, and in patients with cardiovascular diseases, hypertension, obesity, diabetes, chronic pulmonary disease, advanced chronic kidney disease, pregnancy, active solid or hematological cancer, and other immunodeficiency [9–11]. Other apparent predictive factors include laboratory values, for example a high C-reactive protein (CRP) [12]. 

Treatment of hospitalized patients with COVID-19 evolved throughout the pandemic. However, the use of antimicrobial agents was consistently high, with approximately 60% of patients receiving antibiotics, despite the low frequency (≤ 5%) of documented early bacterial coinfection [13–20]. Whether the different epidemic waves (with their own specific epidemiological characteristics, viral variants, and treatment options) are associated with different patient outcomes is yet unclear.

In this study, we aim to address differences in patient characteristics, treatment, and outcome during different epidemic waves of COVID-19 in a Belgian tertiary hospital, and to identify and quantify the effect of different risk factors.

## Methods

### Study design and context

This is an observational study; the registry was started at the start of the pandemic using data from medical and laboratory records. It was conducted at a single tertiary referral hospital, the Antwerp University Hospital in Belgium. The hospital has 573 beds, of which 45 are intensive care unit (ICU) beds. Patients were hospitalized from the emergency department or immediately after transfer from another hospital. Patients were hospitalized on regular medical wards, including temporally dedicated COVID-19 wards, and transferred to the ICU, if necessary, after discussion with an ICU physician, as in routine non-COVID-19 medical care. Treating physicians received guidance in case management through the hospital’s COVID-19 guidance documents formulated by a multidisciplinary team and updated in accordance with Belgian and international guidelines. In particular during peak periods within the pandemic with high pressure on hospital and ICU beds, case management was discussed in a multidisciplinary group to ensure proper patient care and anticipate future ICU admissions. The ICU surge capacity for COVID-19 and non-COVID-19 intensive care unit patients was organized in the ICU and the post-anesthesia care unit, respectively.

### Study period and epidemic waves

**Fig. 1:**
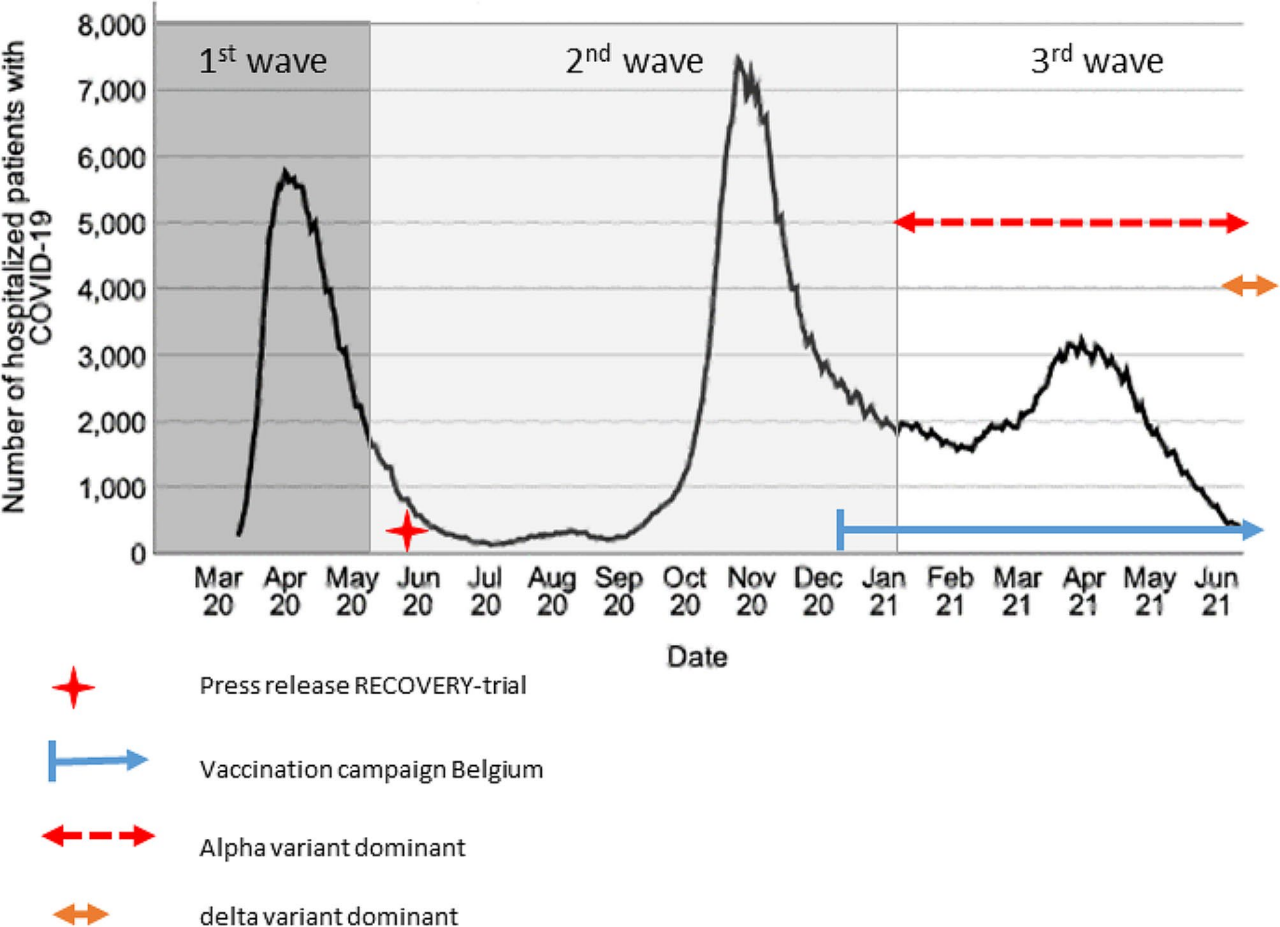
Epidemiologic situation in Belgium during the observation period

Definition of the start and end dates of subsequent epidemic waves was based on national epidemiological data and Antwerp university hospital admission data (Fig. 1) [21]. The first wave was defined as February 29th, 2020, until April 30th, 2020; the second wave, May 1st, 2020 to January 31st, 2021, and the third wave, February 1st, 2021, to June 30, 2021. Nationwide vaccination was initiated December 28, 2020, initially focusing on elderly comorbid people and healthcare providers. By June 30, 2021, at the end of the third wave, a nationwide vaccine coverage of 62% for the first dose and 35% for the second dose was achieved [21]. 

### Eligibility criteria

We included adult patients who were admitted for or developed symptomatic COVID-19 during their hospital stay at the Antwerp University Hospital between February 29, 2020, until June 30, 2021. Both laboratory and radiologically confirmed patients were considered, consistent with the case definition of the Belgian National Institute of Public Health, Sciensano [22]. Criteria for exclusion were hospitalization for a reason other than COVID-19 and, a hospital stay shorter than 24 h.

### Data collection

Data collection was retrospective, but structured medical notes were used for COVID-19 patients during the pandemic. Pseudonymized demographic (age and sex) and clinical patient data were collected from the electronic patient file system and the laboratory data system. Clinical data included date of onset of symptoms and hospital admission, the presence of comorbidities, and CRP. Charlson comorbidity index (CCI) was calculated based on age and the presence of comorbidities [12]. The World Health Organization COVID-19 severity classification was determined at admission to our hospital, categorizing them into non-severe, severe, or critical disease [23]. Different treatments were recorded, including supportive therapies, antimicrobial therapies, and COVID-19 directed medical therapies; as well as the occurrence of complications during hospital stay. The collected outcome measures are ICU admission and hospital mortality.

### Statistical analysis

Statistical analyses were performed using SPSS version 29. Descriptive statistics were used to compare patient characteristics, management, and outcome between the successive epidemic waves. Absolute and relative frequencies were used to analyze categorical and binary variables. Continuous variables (age, CCI, body mass index (BMI), CRP), were categorized and analyzed using absolute and relative frequencies. Differences of the categorical and binary variables were examined with Chi-square test. Length of stay, time to intensive care unit admission, time to death, duration of treatments, and timing of initiation of certain drugs are presented as median (in days) with corresponding interquartile range (IQR). Differences of the continuous variables were examined with Kruskal-Wallis test.

Risk factors for intensive care unit admission and in-hospital death were determined using a multivariable logistic regression, presented as odds ratios (OR) and 95% confidence intervals (CI). Preselected covariates were age, sex, and wave. Other covariates of interest were selected based on statistical significance (*p* < 0.1) in univariable analysis. As we are tertiary care hospital with a lot of tertiary referrals mostly directly to ICU, we described the ICU population in more detail.

### Ethics

The study protocol was approved by the local institutional ethics committee in April 2020 (study number 3461). Informed consent was waived given the retrospective and observational non-interventional nature of this study, focusing on routine medical care.

## Results

In total 722 of 813 adult patients who were admitted with COVID-19 to the Antwerp University Hospital during the observation period were included, 181, 347 and 194 during the first, second and third wave respectively. Excluded patients were not fulfilling the case definition (*n* = 75), hospitalized for less than 24 h (*n* = 2) or readmitted during the observation period (*n* = 14), of which 5 were readmitted for COVID-19.

### Patient characteristics

Table 1 displays the baseline characteristics of patients presenting in the consecutive waves. Patients presenting during the second wave, were significantly older and had more comorbidity. Distribution of CRP upon hospital admission was similar over the successive waves. The timing of hospital admission after symptom onset (*n* = 641) did not change during the first, second and third wave (median 7 days (IQR 4–11), median 7 days (IQR 3–9), median 7 (IQR 4–10), respectively), also disease severity of patients primarily presenting at our hospital did not change over the successive waves.

**Table 1:** Patient characteristics at hospital admission by consecutive epidemic waves

Patient characteristics at hospital admission	All patients (N = 722)	First wave (N = 181)	Second wave (N = 347)	Third wave (N = 194)	p
	n (%)	n (%)	n (%)	n (%)	
Sex					0.320
Female	305 (42.2%)	79 (43.6%)	137 (39.5%)	89 (45.9%)	
Male	417 (57.8%)	102 (56.4%)	210 (60.5%)	105 (54.1%)	
Age, in years					0.006
< 50	144 (19.9%)	30 (16.6%)	64 (18.4%)	50 (25.8%)	
50–65	255 (35.3%)	68 (37.6%)	107 (30.8%)	80 (41.2%)	
65–80	232 (32.1%)	62 (34.3%)	124 (35.7%)	46 (23.7%)	
≥ 80	91 (12.6%)	21 (11.6%)	52 (15.0%)	18 (9.3%)	
Charlson comorbidity index					< 0.001
< 2	293 (40.6%)	109 (60.2%)	97 (28.0%)	87 (44.8%)	
2–4	258 (35.7%)	45 (24.9%)	145 (41.8%)	68 (35.1%)	
≥ 5	171 (23.7%)	27 (15.9%)	105 (30.3%)	39 (20.1%)	
Comorbidities					
Cardiovascular disease	195 (27.0%)	51 (28.2%)	103 (29.7%)	41 (21.1%)	0.092
Hypertension	300 (41.6%)	76 (42.0%)	153 (44.1%)	71 (36.6%)	0.235
Diabetes	158 (21.9%)	35 (19.3%)	87 (25.1%)	36 (18.6%)	0.135
Chronic kidney disease	84 (11.6%)	18 (9.9%)	40 (11.5%)	26 (13.4%)	0.578
Chronic pulmonary disease	132 (18.3%)	35 (19.3%)	62 (17.9%)	35 (18.0%)	0.913
Chronic liver disease	26 (3.6%)	8 (4.4%)	13 (3.7%)	5 (2.6%)	0.620
Neurological condition (not cognitive impairment)	108 (15.0%)	21 (11.6%)	58 (16.7%)	29 (14.9%)	0.295
Cognitive impairment	37 (5.1%)	6 (3.3%)	26 (7.5%)	5 (2.6%)	0.020
Immunodeficiency	86 (11.9%)	38 (21.0%)	34 (9.8%)	14 (7.2%)	< 0.001
Body mass index					0.385
< 25 kg/m²	157 (21.7%)	45 (24.9%)	71 (20.5%)	41 (21.1%)	
25–30 kg/m²	209 (28.9%)	58 (32.0%)	99 (28.5%)	52 (26.8%)	
> 30 kg/m²	175 (24.2%)	35 (19.3%)	84 (24.2%)	56 (28.9%)	
Missing values	181 (25.1%)	43 (23.8%)	93 (26.8%)	45 (23.2%)	
COVID-19 vaccination (at least one dose prior to hospital admission)	20 (2.8%)	0 (0.0%)	0 (0.0%)	20 (10.3%)	< 0.001
Transfer from another hospital	118 (16.3%)	10 (5.5%)	67 (19.3%)	41 (21.1%)	< 0.001
Nosocomial	39 (5.4%)	20 (11.0%)	14 (4.0%)	5 (2.6%)	< 0.001
WHO COVID-19 classification at hospital admission*					
Non-severe	468 (81.4%)	127 (83.0%)	221 (81.9%)	120 (78.9%)	0.735
Severe	58 (10.1%)	16 (10.5%)	24 (8.9%)	18 (11.8%)	
Critical	49 (8.5%)	10 (6.5%)	25 (9.3%)	14 (9.2%)	
C-reactive protein					0.919
CRP < 50 mg/l	266 (36.8%)	71 (39.2%)	127 (36.6%)	68 (35.1%)	
CRP 50–150 mg/l	286 (39.6%)	72 (39.8%)	136 (39.2%)	78 (40.2%)	
CRP ≥ 150 mg/l	159 (22.0%)	38 (21.0%)	74 (21.3%)	47 (24.2%)	
Missing values	11 (1.5%)	0 (0.0%)	10 (2.9%)	1 (0.5%)	

Distribution of treatments characteristics at hospital admission of patients with COVID-19 by consecutive epidemic wave, presented as absolute frequencies and proportions

*Only for non transferred, non nosocomial (n = 575)

### Inpatient medical management

Overall, 83% received oxygen therapy, for a median duration of 3 days (IQR 2–7) (Table 2). An increasing use of noninvasive ventilation, mostly high-flow oxygen therapy, was observed over the subsequent waves. This increase was observed in the ICU after the first wave, and in patients who were never transferred to the ICU, respectively in 1%, 3%, and 9% of all patients in the first, second and third wave. During the first wave, hydroxychloroquine was used as an antiviral and immunomodulatory agent in 77% of patients; this practice was completely abandoned thereafter. Corticosteroids were occasionally used during the first wave, but were initiated later than in subsequent waves, 9 (4–14), 1 (0–3), and 1 (0–3) days (IQR) after hospital admission during the first, second and third wave respectively. Remdesivir was the only used antiviral agent in this stage of the pandemic, but its use was also limited (2.5% (18/722)) and abandoned after the second wave. We observed a decrease in antibiotic prescriptions at hospital admission (excluding patients transferred from another hospital) from 55% (94/171) during the first wave to less than 30% (44/153) in the third wave.

**Table 2:** Inpatient management by consecutive epidemic waves

Management	All patients (N = 722)	First wave (N = 181)	Second wave (N = 347)	Third wave (N = 194)	p
	n (%)	n (%)	n (%)	n (%)	
Intensive care unit admission	225 (31.2%)	48 (26.5%)	115 (33.1%)	62 (32.0%)	0.285
Oxygen therapy (nasal prongs or mask)	601 (83.2%)	158 (87.3%)	282 (81.3%)	161 (83.0%)	0.112
Non-invasive ventilation (including high-flow oxygen therapy)	156 (21.6%)	23 (12.7%)	76 (21.9%)	57 (29.4%)	< 0.001
non-ICU	30 (4.1%)	2 (1.1%)	10 (2.9%)	18 (9.3%)	
ICU	126 (21.6%)	21 (11.6%)	66 (19.0%)	39 (20.1%)	
Invasive ventilation	165 (22.9%)	42 (23.2%)	75 (21.6%)	48 (24.7%)	0.780
Prone ventilation	95 (13.2%)	22 (12.2%)	37 (10.7%)	36 (18.6%)	0.035
Tracheostomy	59 (8.2%)	11 (6.1%)	27 (7.8%)	21 (10.8%)	0.242
Extra-corporeal membrane oxygenation	33 (4.6%)	5 (2.8%)	12 (3.5%)	16 (8.2%)	0.025
Renal replacement therapy	31 (4.3%)	8 (4.4%)	16 (4.6%)	7 (3.6%)	0.844
Hydroxychloroquine	140 (19.4%)	140 (77.3%)	0 (0.0%)	0 (0.0%)	< 0.001
Remdesivir	18 (2.5%)	0 (0.0%)	18 (5.2%)	0 (0.0%)	< 0.001
Corticosteroids	394 (54.6%)	39 (21.5%)	224 (64.6%)	131 (67.5%)	< 0.001
> 1 course	57 (7.9%)	0 (0.0%)	24 (6.9%)	33 (17.0%)	< 0.001
Antibiotic start at hospital admission	303 (42.0%)	102 (56.4%)	131 (37.8%)	70 (36.1%)	< 0.001
Antifungals	69 (9.6%)	12 (6.6%)	33 (9.5%)	24 (12.4%)	0.425
Immunomodulators for COVID-19	13 (1.8%)	1 (0.6%)	12 (3.5%)	0 (0.0%)	0.020
COVID-19 convalescent plasma	5 (0.7%)	0 (0.0%)	3 (0.9%)	2 (1.0%)	0.418
**Antibiotic use in non-transferred patients**	**All patients** **(N = 604)**	**First wave** **(N = 171)**	**Second wave** **(N = 280)**	**Third wave** **(N = 153)**	**p**
	**n (%)**	**n (%)**	**n (%)**	**n (%)**	
Antibiotic start at hospital admission	238 (39.4%)	94 (55.0%)	100 (35.7%)	44 (28.8%)	< 0.001
Product					0.027
Amoxicillin-clavulanic acid	190 (79.8%)	69 (73.4%)	86 (86.0%)	35 (79.5%)	
Piperacillin-tazobactam	22 (9.2%)	12 (12.8%)	3 (3.0%)	7 (15.9%)	
Other	26 (10.9%)	13 (13.8%)	11 (11.0%)	2 (4.5%)	

Distribution of treatments used for inpatient management of patients with COVID-19 by consecutive epidemic wave, presented as absolute frequencies and proportions

### Outcome and complications

We observed an increase in complications over the different waves, including acute respiratory distress syndrome (ARDS), thrombo-embolic disease, ventilator associated pneumonia, and neurologic complications (Table 3). One hundred seven patients died because of COVID-19 or its complications. The overall in-hospital mortality decreased across the waves, although not statistically significant, mostly driven by a decreased mortality in non-ICU patients; 16%, 8%, and 5% during the first, second and third wave respectively (*p* = 0.007). Patients died after a median duration of 8 days after hospital admission (IQR 5–14) on a regular medical ward, and 20 days (IQR 14–38) for ICU patients. Median length of hospital stay was 9 days (IQR 5–19), but 27 days (IQR 16–47) in patients admitted to the intensive care unit.

**Table 3:** Outcomes by consecutive epidemic waves using descriptive statistics

Outcome	All patients (N = 722)	First wave (N = 181)	Second wave (N = 347)	Third wave (N = 194)	p
	n (%)	n (%)	n (%)	n (%)	
Hospital mortality	107 (14.8%)	30 (16.6%)	54 (15.6%)	23 (11.9%)	0.378
Complications					
Acute respiratory distress syndrome	149 (20.6%)	29 (16.0%)	72 (20.7%)	48 (24.7%)	0.005
Multi-organ failure	38 (5.3%)	5 (2.8%)	17 (4.9%)	16 (8.2%)	0.014
Septic shock	46 (6.4%)	12 (6.6%)	25 (7.2%)	9 (4.6%)	0.034
Severe acute kidney injury	115 (15.9%)	16 (8.8%)	62 (17.9%)	37 (19.1%)	0.013
Myocarditis	3 (0.4%)	2 (1.1%)	1 (0.3%)	0 (0.0%)	0.170
Arrhythmia	96 (13.3%)	26 (14.4%)	46 (13.3%)	24 (12.4%)	0.847
Pneumothorax	17 (2.4%)	1 (0.6%)	9 (2.6%)	7 (3.6%)	0.140
COVID-19 associated pulmonary aspergillosis	22 (3.0%)	7 (3.9%)	5 (1.4%)	10 (5.2%)	0.052
Thromboembolic disease (including ischemic stroke)	59 (8.2%)	8 (4.4%)	28 (8.1%)	23 (11.9%)	0.003
Hospital acquired pneumonia / ventilator associated pneumonia	129 (17.9%)	23 (12.7%)	61 (17.6%)	45 (23.2%)	0.029
Bloodstream infection	56 (7.8%)	17 (9.4%)	24 (6.9%)	15 (7.7%)	0.393
Neurological sequelae (non-stroke)	92 (12.7%)	24 (13.3%)	44 (12.7%)	24 (12.4%)	0.027
Bleeding	47 (6.5%)	2 (1.1%)	32 (9.2%)	13 (6.7%)	< 0.001
	Median [IQR]	Median [IQR]	Median [IQR]	Median [IQR]	p
Length of hospital stay (days)	9 (5–19)	9 (5–21)	10 (5–18)	8 (4–20)	0.476
Intensive care unit patients (days)	27 (15.5–46.5)	33 (18–56)	22 (14–43)	32 (18–50)	0.025
Length of intensive care unit stay (days)	18 (8–37)	18 (12–35)	15 (6–34)	25 (11–47)	0.040
Time to intensive care unit (days)	0 (0–2)	1 (0–3)	0 (0–3)	0 (0–1)	0.015
Non-transferred patients (days)	1 (0–4)	2 (1–4)	1 (0–5)	1 (0–3)	0.222
Time to death (days)	15 (7–28)	12 (6–22)	15 (8–28)	18 (8–43)	0.340
Normal ward (days)	8 (5–14.25)	8 (5–15)	8 (4–11)	8 (5–15)	0.667
Intensive care unit (days)	20 (13.5–38)	21 (11–30)	19 (14–31)	21 (17–44)	0.724

Distribution of outcomes by consecutive epidemic waves presented as absolute frequencies and proportions

Comparison between three consecutive waves of length of stay, length of intensive care unit stay, time to intensive care unit admission, and time to death expressed as median days and with corresponding interquartile range (IQR)

### Characteristics, management and outcome of patients admitted to the ICU

31% of all patients were admitted to the ICU (Table 4). Almost half of the patients were older than 65 years old with fewer people having a CCI < 2 (34% vs. 40% in all patients admitted to our hospital). More than one in three patients admitted to our ICU was transferred from another hospital. Intubation and invasive ventilation were necessary in 73%, with a median number of invasive ventilation days of 20 (IQR 13–39). 20% of patients received more than one course of corticosteroids, the second course being an additional treatment for ARDS. Besides respiratory and infectious complications, we observed neurological sequelae (non-stroke) in one third of ICU patients. Most of these were ICU acquired weakness (critical illness polyneuropathy). Length of ICU stay was shorter during the second wave (median 15 days, IQR 6–34), compared to the first wave (median 18 days, IQR 12–35) and third wave (median 23 days, IQR 11–51). Median length of stay was 49 days (IQR 31–70) in patients with neurological sequelae, compared to 18 days (IQR 12–32) in patients without neurological sequelae.

**Table 4:** Clinical characteristics at hospital admission and management of intensive care unit patients

Characteristics of ICU patients	Intensive care unit patients (N = 225)
	n (%)
Clinical characteristics	
Sex	
Female	79 (35.1%)
Male	146 (64.9%)
Age, in years	
< 50	31 (13.8%)
50–65	89 (39.6%)
65–80	98 (43.6%)
≥ 80	7 (3.1%)
Charlson comorbidity index	
< 2	77 (34.2%)
2–4	108 (48.0%)
≥ 5	40 (17.8%)
Comorbidities	
Cardiovascular disease	57 (25.3%)
Hypertension	108 (48.0%)
Diabetes	56 (24.9%)
Chronic kidney disease	26 (11.6%)
Chronic pulmonary disease	37 (16.4%)
Chronic liver disease	11 (4.9%)
Neurological condition (not cognitive impairment)	27 (12.0%)
Cognitive impairment	4 (1.8%)
Immunodeficiency	20 (8.9%)
COVID-19 vaccination	5 (2.2%)
Body mass index	
< 25 kg/m²	59 (26.2%)
25–30 kg/m²	86 (38.2%)
> 30 kg/m²	73 (32.4%)
Missing values	7 (3.1%)
Initial WHO COVID-19 classification at hospital admission	
Non-severe	78 (34.7%)
Severe	24 (10.7%)
Critical	123 (54.7%)
C-reactive protein at hospital admission	
CRP < 50 mg/l	47 (20.9%)
CRP 50–150 mg/l	84 (37.3%)
CRP ≥ 150 mg/l	92 (40.9%)
Missing values	2 (0.9%)
C-reactive protein at intensive care unit admission	
CRP < 50 mg/l	32 (14.2%)
CRP 50–150 mg/l	76 (33.8%)
CRP ≥ 150 mg/l	115 (51.1%)
Missing values	2 (0.9%)
Transfer	85 (37.8%)
Management of ICU patients	
Non-invasive ventilation (including high-flow oxygen therapy)	126 (56.0%)
Invasive ventilation	165 (73.3%)
Prone ventilation	95 (42.2%)
Tracheostomy	58 (25.8%)
Extra-corporeal membrane oxygenation	33 (14.7%)
Renal replacement therapy	30 (13.3%)
Hydroxychloroquine	42 (18.7%)
Remdesivir	10 (4.4%)
Corticosteroids	177 (78.7%)
> 1 regimen	45 (20.0%)
Antibiotic start at hospital admission	138 (61.3%)
Antifungals	64 (28.4%)
Immunomodulators for COVID-19	11 (4.9%)
Convalescent plasma	5 (2.2%)
Outcome of ICU patients	
Hospital mortality	61 (27.1%)
Complications	
Acute respiratory distress syndrome	138 (61.3%)
Multi-organ failure	22 (9.8%)
Septic shock	43 (19.1%)
Severe acute kidney injury	57 (25.3%)
Myocarditis	2 (0.9%)
Arrhythmia	63 (28.0%)
Pneumothorax	17 (7.6%)
COVID-19 associated pulmonary aspergillosis	19 (8.4%)
Thromboembolic disease (including ischemic stroke)	35 (15.6%)
Hospital acquired pneumonia / ventilator associated pneumonia	123 (54.7%)
Bloodstream infection	43 (19.1%)
Neurological sequelae (non-stroke)	78 (34.7%)
Bleeding	36 (16.0%)

Distribution of clinical characteristics at hospital admission, management, and outcome of intensive care unit patients, presented as absolute frequencies and proportions

### Risk factors for ICU admission and hospital mortality

Cases occurring during the second (OR 0.30, 95% CI 0.18–0.52) and third wave (OR 0.24, 95% CI 0.13–0.44) had a decreased risk of death, though no differences in risk for ICU admission were found (Table 5). In our population increasing age above 50 years old was found protective against mortality, on the other hand patients above 80 years old were less likely to be admitted to ICU. We found that patients with a CCI ≥ 5 were less likely to be admitted to the ICU but at a higher risk of dying in the hospital. Both for admission to ICU as well as hospital mortality having a BMI above 25 kg/m² was protective. High CRP at hospital admission was a risk factor for ICU admission (OR 3.59, 95% CI 2.26–5.72) in our cohort, but was not associated with death. Transfers from another hospital were associated with mortality during hospital stay. Comparable results were found after excluding nosocomial, and transferred patients (supplementary Table S1).

**Table 5:** Associations between different potential risk factors and outcome

Multivariable logistic regression	ICU admission *p* < 0.001	p	Death *p* < 0.001	p
	OR [95% CI]		OR [95% CI]	
Epidemic wave				
First wave		0.505		< 0.001
Second wave	0.95 (0.61–1.47)	0.819	0.30 (0.17–0.53)	< 0.001
Third wave	0.77 (0.48–1.23)	0.268	0.26 (0.14–0.48)	< 0.001
Age				
< 50		0.001		0.001
50–65	0.77 (0.46–1.27)	0.297	0.23 (0.12–0.47)	< 0.001
65–80	1.02 (0.55–1.89)	0.945	0.37 (0.17–0.80)	0.012
≥ 80	0.17 (0.06–0.46)	0.001	0.36 (0.13–0.95)	0.038
Sex				
Female				
Male	1.20 (0.83–1.73)	0.343	0.71 (0.44–1.14)	0.155
Charlson comorbidity index				
< 2		0.035		< 0.001
2–4	1.03 (0.62–1.71)	0.903	1.46 (0.71–2.99)	0.303
≥ 5	0.53 (0.28–0.99)	0.048	5.68 (2.54–12.70)	< 0.001
Body mass index				
< 25 kg/m²		0.029		< 0.001
25–30 kg/m²	0.60 (0.39–0.92)	0.020	0.54 (0.33–0.89)	0.016
> 30 kg/m²	0.60 (0.38–0.94)	0.025	0.28 (0.15–0.51)	< 0.001
C-reactive protein				
CRP < 50 mg/l		< 0.001		0.084
CRP 50–150 mg/l	1.44 (0.95–2.19)	0.088	1.11 (0.65–1.89)	0.708
CRP ≥ 150 mg/l	3.77 (2.32–6.15)	< 0.001	1.92 (1.05–3.52)	0.034
Transfer				
	N/A		3.78 (2.05–6.98)	< 0.001

Associations between different potential risk factors and outcome and the following outcomes: intensive care unit admission, and death, expressed as odds ratios (OR) with 95% confidence interval (CI) obtained by multivariable logistic regression

## Discussion

This retrospective observational study of COVID-19 patients in a Belgian university hospital provides us with an overview and comparison of patient characteristics and management during the first three consecutive epidemic waves. We found demographic differences between the waves, patients being older and with more comorbidities in the second wave. In ICU an increased number of transfers for tertiary care was observed across the waves, as well as more organ failure and increased ECMO use, reflecting an increasing selection of the most critically ill patients in a tertiary care hospital such as ours.

Our hospitalized patient population with SARS-Cov2 infection is comparable to other Belgian hospitals, with male predominance and 80% patients older than 50 years old [23, 24]. The proportion of patients admitted to ICU is slightly higher (31% vs. 25%), reflecting the tertiary referral function and the relative high proportion of ICU-beds in our hospital (8% of all beds), and is also higher than in multinational studies such as ISARIC (15.3% ICU admission) [25]. In spite of the hospital’s tertiary function, COVID-19 related mortality was lower than the Belgian in-hospital average during the first wave (17% vs. 21%) [23], and multinational data for first and second waves (21%) [25]. Moreover, although the third wave patients were probably infected with the more virulent Alpha variant [1, 2], outcome was better than in the preceding epidemic waves. It remains difficult to compare intensive care unit outcome data as criteria for intensive care unit admission differ significantly between centers and countries, and the influence of the pandemic pressure on ICU [26]. 

During the third wave the nationwide vaccination campaign started, prioritizing elderly and comorbid people, and reaching a vaccine uptake of two thirds for a first dose and one third of Belgian citizens received a second dose by the end of the third wave [24]. Vaccination with at least one dose was only reported in 10% of patients admitted during the third wave, while we observed a clear decrease in admission of patients older than 65 years with comorbidities.

Between the first and second waves, we noted a significant change in case management, including the abolishment of hydroxychloroquine use and widespread use of corticosteroids early in the disease course. Furthermore, we saw an increased use of non-invasive ventilation (mainly high-flow nasal oxygen therapy) after the first wave. This practice was also adopted on regular wards: before ICU transfer, step down from ICU and for patients who were considered poor candidates for intensive care unit admission. Another evolution in case management was the decrease in antibiotic prescriptions at hospital admission, which was the result of growing knowledge on the low incidence of early bacterial co- or superinfection and ongoing antimicrobial stewardship [14]. 

An intriguing observation in our cohort is that age is inversely associated with death. This is opposite to the findings in other studies [9–11, 23, 25]. We cannot completely explain this finding, probably several factors are contributing. The small sample size, and the fact that the analysis was carried out in a hospital with tertiary referral function might be an explanation for this finding, with younger critical COVID patients undergoing ECMO treatment as well as admitting patients treated in our tertiary care center (complex hematology and oncology, solid-organ transplant patients). As seen in Table 5, not only very old patients, but also patients with CCI of at least 5 were less likely admitted to ICU. This can probably be explained by the fact that these patients had more advanced care planning decisions with limitations in therapy such as mechanical ventilation during the multidisciplinary meetings. Their poorer prognosis in COVID 19, was noted in several studies reviewed in a meta-analysis in 2020 showing an increase in mortality by 16% with each in CCI by one point [27]. This might have led to a particular case mix where analysis of risk factors is different from analysis in more general population.

The strengths of our study include the clinical and treatment information, available for all individuals admitted for COVID-19 since the start of the pandemic until the end of the third epidemic wave. Furthermore, it involves real world data from a single center, allowing to compare influence of different demographics, virus variants and case management between the respective waves. Therefore, our data offer a detailed insight in the evolution of the patient population and the clinical impact of COVID-19 during the first pandemic year. Despite the retrospective design, data collection was standardized and of high quality (few missing data) during the entire study period. Our results are however specific for our regional setting, and not necessarily generalizable to other (similar) socio-geographical regions [28, 29]. Healthcare and policy may differ, as well as circulating infectious strains, uptake of vaccinations and infection control measurements, and preparedness. Therefore, we do strongly advocate for widespread data collection and analyses, to facilitate data-driven improvements in healthcare, and to be better prepared for future emergencies.

## Conclusions

Despite increasing disease severity and more comorbid patients, mortality decreased each subsequent wave. Evolutions in patient management such as high-flow oxygen therapy on regular wards and corticosteroid use may explain this finding.

### Electronic supplementary material

Below is the link to the electronic supplementary material.

**Supplementary Table 1** Associations between different potential risk factors and outcome after exclusion of nosocomial infected patients and transferred patients.

## Data Availability

Upon reasonable request and after required approval from Ethics Committee.

## Abbreviations

CRP: C-reactive protein
ICU: intensive care unit
CCI: Charlson comorbidity index
BMI: body mass index
OR: odds ratio
CI: confidence interval
IQR: interquartile range
ECMO: extracorporeal membrane oxygenation
ARDS: acute respiratory distress syndrome
