# Supplementary material for: COVID-19 in three waves in a tertiary referral hospital in Belgium: a comparison of patient characteristics, management, and outcome
Source: Virol J. 2024 May 30;21:119. doi: 10.1186/s12985-024-02360-8 (PMC11138039; doi:10.1186/s12985-024-02360-8)
Supplement: Supplementary file 1 — Supplementary Table 1 Associations between different potential risk factors and outcome after exclusion of nosocomial infected patients and transferred patients. [file 12985_2024_2360_MOESM1_ESM.docx]

Supplementary table S1: Associations between different potential risk factors and outcome after exclusion of nosocomial, and transferred patients

| Multivariable logistic regression | | ICU admission  p<0.001 |  | Death  p<0.001 |  |
| --- | --- | --- | --- | --- | --- |
|  | | OR [95% CI] | p | OR [95% CI] | p |
| Epidemic wave | |  |  |  |  |
|  | First wave |  | 0.004 |  | <0.001 |
|  | Second wave | 0.603 (0.306 - 1.009) | 0.054 | 0.241 (0.117 - 0.494) | <0.001 |
|  | Third wave | 0.377 (0.210 - 0.678) | 0.001 | 0.259 (0.118 - 0.568) | 0.001 |
| Age |  |  |  |  |  |
|  | < 50 |  | 0.001 |  | 0.002 |
|  | 50 - 65 | 0.707 (0.389 - 1.286) | 0.256 | 0.157 (0.061 - 0.402) | <0.001 |
|  | 65 - 80 | 1.436 (0.704 - 2.927) | 0.319 | 0.341 (0.132 - 0.881) | 0.026 |
|  | ≥ 80 | 0.252 (0.086 - 0.740) | 0.012 | 0.372 (0.121 - 1.145) | 0.085 |
| Sex |  |  |  |  |  |
|  | Female |  |  |  |  |
|  | Male | 1.030 (0.663 - 1.599) | 0.896 | 0.954 (0.525 - 1.733) | 0.876 |
| Charlson comorbidity index | |  |  |  |  |
|  | < 2 |  | 0.780 |  | <0.001 |
|  | 2 - 4 | 0.941 (0.513 - 1.725) | 0.844 | 1.376 (0.548 - 3.452) | 0.497 |
|  | ≥ 5 | 0.775 (0.363 - 1.657) | 0.511 | 6.189 (2.236 - 17.128) | <0.001 |
| Body mass index | |  |  |  |  |
|  | < 25 kg/m² |  | 0.007 |  | <0.001 |
|  | 25 - 30 kg/m² | 0.482 (0.291 - 0.798) | 0.005 | 0.486 (0.265 - 0.891) | 0.020 |
|  | > 30 kg/m² | 0.512 (0.302 - 0.869) | 0.013 | 0.209 (0.091 - 0.477) | <0.001 |
| C-reactive protein | |  |  |  |  |
|  | CRP < 50 mg/l |  | <0.001 |  | 0.505 |
|  | CRP 50 - 150 mg/l | 1.534 (0.927 - 2.540) | 0.096 | 1.138 (0589 - 2.198) | 0.700 |
|  | CRP ≥ 150 mg/l | 4.863 (2.651 - 8.924) | <0.001 | 1.630 (0.715 - 3.714) | 0.245 |

Associations between different potential risk factors and outcome after exclusion of nosocomial, and transferred patients and for the following outcomes: intensive care unit admission, and death, expressed as odds ratios (OR) with 95% confidence interval (CI) obtained by multivariable logistic regression.
